# Lipid oxidation dysregulation: an emerging player in the pathophysiology of sepsis

**DOI:** 10.3389/fimmu.2023.1224335

**Published:** 2023-08-03

**Authors:** Renan Muniz-Santos, Giovanna Lucieri-Costa, Matheus Augusto P. de Almeida, Isabelle Moraes-de-Souza, Maria Alice Dos Santos Mascarenhas Brito, Adriana Ribeiro Silva, Cassiano Felippe Gonçalves-de-Albuquerque

**Affiliations:** ^1^ Laboratory of Immunopharmacology, Department of Physiology, Federal University of the State of Rio de Janeiro, Rio de Janeiro, Brazil; ^2^ Neuroscience Graduate Program, Federal Fluminense University, Niteroi, Brazil; ^3^ Laboratory of Immunopharmacology, Oswaldo Cruz Institute, Oswaldo Cruz Foundation, Rio de Janeiro, Brazil

**Keywords:** fatty acid oxidation (FAO), inflammation, metabolic dysfunction, free fatty acid (FFA), sepsis

## Abstract

Sepsis is a life-threatening organ dysfunction caused by abnormal host response to infection. Millions of people are affected annually worldwide. Derangement of the inflammatory response is crucial in sepsis pathogenesis. However, metabolic, coagulation, and thermoregulatory alterations also occur in patients with sepsis. Fatty acid mobilization and oxidation changes may assume the role of a protagonist in sepsis pathogenesis. Lipid oxidation and free fatty acids (FFAs) are potentially valuable markers for sepsis diagnosis and prognosis. Herein, we discuss inflammatory and metabolic dysfunction during sepsis, focusing on fatty acid oxidation (FAO) alterations in the liver and muscle (skeletal and cardiac) and their implications in sepsis development.

## 1 Introduction

Sepsis is a Greek-derived term that initially meant “decomposition of animal or vegetable organic matter in the presence of bacteria.” This has been documented in Homer’s Iliad and Corpus Hippocraticum (1). Over the last 120 years, sepsis has received different modern definitions, most related to the interaction between pathogenic microorganisms and immune host defenses (2–5). Sepsis is defined “as life-threatening organ dysfunction caused by a dysregulated host response to infection” (6).

Sepsis is the most common cause of death by multiple organ dysfunction in critically ill patients, and, as an infectious condition, sepsis can be bacterial, viral, or fungal (7–9). Sepsis affects 1.7 million adults annually in the United States, and half of all sepsis cases are caused by gram-negative bacteria, making septic shock a common complication (10, 11). In addition, gram-positive bacteria cause septic shock. We also must consider that many studies use lipopolysaccharide (LPS), a component of gram-negative bacteria, which causes endotoxemia, but not sepsis, but can be biased considering gram-negative sepsis. More than 40 million annual cases are estimated to occur worldwide (12). Detecting the origin of sepsis is crucial in clinical practice because it presents different pathophysiologies, clinical patterns, and treatment approaches (13, 14).

To assess sepsis severity, the Sequential Organ Failure Assessment (SOFA) score evaluates six organ systems: the respiratory, cardiovascular, hepatic, coagulation, renal, and neurological systems (15, 16). Dysregulation of lipid oxidation plays a significant role in organ damage during sepsis, and studies have indicated a positive correlation between markers of lipid oxidation dysfunction and SOFA score. Serum malondialdehyde (MDA) and free fatty acids (FFA) levels also correlated with higher SOFA scores (17). For example, serum MDA is a marker of lipid peroxidation and oxidative stress that correlates with SOFA score and lactatemia (18). Recent research has also shown connections between MDA levels and SOFA score, as well as with APACHE-II and coagulation indices (19). In addition, a correlation between higher FFA levels and patient SOFA scores has been reported (20). Recent research has identified a correlation between SOFA score and various lipid metabolites involved in different pathways (21). Lipid metabolism dysfunction can cause muscle and liver damage but can also affect other systems evaluated by the SOFA score.

Inflammation is fundamental to sepsis pathogenesis but does not fully explain progressive organ dysfunction (22). In addition to inflammation, there are also reported impairments in metabolism, coagulation, and thermoregulation have also been reported (23). Mild alterations in metabolic pathways can be beneficial for regulating the immune response to infection and minimizing tissue damage (24). In sepsis, there is an interplay between inflammatory and metabolic changes, where the host response to infection plays a crucial role, potentially contributing to multiple organ dysfunction and, in severe cases, death (25). Recognizing the interdependency of inflammation and metabolism can be vital, as exploring metabolic alterations and inflammation may unveil new targets for sepsis treatment, which are currently primarily supportive rather than curative (26).

Mitochondrial or energetic dysfunction is among the most widely acknowledged metabolic impairments in sepsis and contributes to organ dysfunction and mortality (27, 28). As fatty acid oxidation (FAO) and the Krebs cycle are intramitochondrial events, a thorough exploration of lipid metabolism in sepsis has been conducted (29). As changes in fatty acid mobilization and oxidation are thought to play a role in the complex pathogenesis of sepsis, they hold significant promise for supporting the development of novel prognostic, therapeutic, and diagnostic tools (30).

Several investigations, including “-omics” and “multi-omics” analyses, have been performed to understand sepsis-induced FAO impairment, analyzing its consequences, predictive tools, and new therapeutic target compounds (31–36). Diverse metabolic adaptations occur in different organs during sepsis and some events can hamper these adaptations (22). For example, sepsis disturbances in lipid metabolism impair β-oxidation, which can lead to lipotoxicity, worsening the clinical status of these patients (37). To explore recent developments in our understanding of lipid metabolism in sepsis, we discuss the impairment of FFA metabolism induced by bacterial sepsis in the liver and muscles, including the skeletal and cardiac systems.

## 2 Sepsis inflammatory response

The latest international consensus (Sepsis-3) defines sepsis as life-threatening organ dysfunction caused by an abnormal host response to an infectious insult (6). Dysfunctional inflammatory and anti-inflammatory responses in sepsis may cause self-damage to the host organs (38). This is often observed in septic patients with acute respiratory failure, renal failure, or circulatory shock (39).

Sepsis can result from systemic inflammatory response syndrome (SIRS), which is caused by the release of pathogen-associated molecular patterns (PAMPs), such as LPS, peptidoglycans, viral ss/dsRNA, and bacterial DNA (40, 41). These molecules are recognized by pattern recognition receptors (PRRs) such as C-type lectin receptors (CLRs), NOD-like receptors (NLRs), and Toll-like receptors (TLRs) (42). To date, 13 TLRs have been described in mammals; ten are expressed in human cells. The activation of this receptor by its ligands triggers intracellular signaling by adaptor proteins; for instance, the MyD88-dependent pathway triggers several kinase proteins, such as p38 MAP kinase, JNK, ERK1, and ERK2, through phosphorylation. It culminates in activating transcription factors (e.g., NF-κB and AP-1) and the production of proinflammatory cytokines such as TNF-α, IL-6, IFN-γ, and enzymes such as cyclooxygenase-2 (COX-2) (43–45). Indeed, using the cecal ligation and puncture (CLP) sepsis model, TLR4 activation increased TNF-α serum levels as early as 4 h after surgery (46).

NOD-like receptors (NLRs) are important PRRs involved in sepsis caused by bacterial infections. They can detect PAMPs and DAMPs (41). NLR proteins form inflammasomes, which are multiprotein complexes (47, 48). Inflammasomes consist of NLR, the adaptor protein apoptosis-associated speck-like protein with a CARD domain (ASC), and pro-caspase-1, which activates caspase-1 and releases IL-1β and IL-18 (47).

The NLRP3 inflammasome modulates responses to sepsis by activation when cells are primed with cytokines or LPS and then stimulated with ATP, reactive oxygen species (ROS), mitochondrial dysfunction, or K^+^ efflux (49). Inhibition of the NLRP3 inflammasome during sepsis can improve survival and bacterial clearance (50), and alleviate acute lung injury. However, it may impair the immune response of monocytes, leading to higher mortality (51, 52). Higher levels of NLRP3, caspase-1, and IL-1 β in the bloodstream of patients with sepsis increase the risk of mortality (53–55).

PRRs activate cytokines that regulate the immune system and contribute to inflammatory responses (56). Although the expression of cytokines is beneficial in the inflammatory response and is necessary for pathogen clearance, excessive cytokine production during sepsis can cause harm, known as a cytokine storm (CS) or cytokine release syndrome (CRS). High levels can lead to organ failure, blood clotting, and even death. (57, 58). For example, in sepsis, cytokines such as IL-6, TNF-α, IL-1β, and CXCL8 increase antibody production, vascular permeability, and neutrophil recruitment (57, 59).

The altered state of endothelial cells, leukocytes, and platelets caused by cytokine production may cause sepsis-induced coagulopathy (SIC), which is linked to a poor outcome (60, 61). During sepsis, monocytes/macrophages express tissue factor and factor VII, activating the extrinsic coagulation pathway. Platelets are also activated, contributing to inflammation and thrombosis. Activated platelets can interact with leukocytes, promoting cytokine release and the neutrophils’ extracellular traps (NETs) (61–64).

Sepsis affects endothelial cells, causing an increase in vascular permeability and the expression of adhesion molecules such as E- and P-selectins, vascular adhesion molecule (VCAM)-1, and intracellular adhesion molecule (ICAM)-1, fostering leucocyte rolling and crawling. Damage to endothelial cells also impairs vascular tonus due to dysregulated NO production. These changes can lead to edema and impaired vascular tonus, compromising organ perfusion and metabolic function (65–69).

Under homeostatic conditions, the ATP demands of immune cells are regulated by the tricarboxylic acid (TCA) cycle (70). Sepsis rapidly increases ATP burning as immune cells prompt the fight against infection. Thus, these cells switch to aerobic glycolysis, quickly producing lactate and ATP but less efficiently. Interestingly, lactate is elevated in sepsis, showing a metabolic turnover in these patients (71), and has also been suggested to be a reliable prognostic tool that helps in the clinical management of patients (71). During sepsis, macrophages produce NO, which inhibits succinate dehydrogenase, a crucial enzyme in the TCA cycle (72). Also in sepsis, M2 macrophages increase lipid β-oxidation and upregulate IL-4 and peroxisome proliferator-activator receptor-γ (PPAR-γ) (72, 73).

## 3 Stressful state of sepsis

Sepsis is a sustained and extreme example of a stressful situation. Stress is “a state of homeostasis being challenged” (74). The stress system has two types of effectors: central and peripheral. The central system corresponds to the hypothalamic hormones arginine vasopressin (AVP), corticotropin-releasing hormone (CRH), pro­opiomelanocortin-derived peptides, and norepinephrine. The peripheral system includes glucocorticoids (the hypothalamic–pituitary–adrenal axis) and catecholamines (the sympathetic–adrenal–medullar axis). All these stress mediators reach the CNS and peripheral functions commanded by the gastrointestinal, cardiorespiratory, metabolic, and immune systems (75). PAMPs and DAMPs excessively activate PPRs, which activate proinflammatory signaling pathways, leading to the release of proinflammatory chemokines, cytokines, and the expression of endothelial adhesion molecules (76). Proinflammatory cytokines, such as TNF-α, IL-1-β, and IL-6, activate the hypothalamic–pituitary–adrenal (HPA) and sympathetic–adrenal–medullar axes (SAM) (77, 78). Activating these axes enhances the synthesis and release of glucocorticoids and catecholamines, thereby promoting metabolic alterations.

The HPA axis controls glucocorticoid and mineralocorticoid syntheses. Glucocorticoids (GCs) are steroid hormones that exert widespread hormone-physiological effects on homeostasis (79). Under internal and external signals, the hypothalamus secretes corticotropin-releasing hormone (CRH) and arginine vasopressin (AVP) (80). CRH is released into the hypophyseal portal circulation and stimulates the anterior pituitary to produce and secrete adrenocorticotropic hormone (ACTH). Consequently, glucocorticoids (cortisol in humans and corticosterone in rodents) are released from the zona fasciculata of the adrenal cortex (81). GCs are transported through the globulin-glucocorticoid protein in the blood. The glucocorticoid receptor (GR), which is ubiquitously expressed throughout the organism, mediates the glucocorticoid effects. The GC–GR complex translocates into the nucleus and modulates target gene expression through glucocorticoid-responsive elements (GREs) (82). Owing to hyperglucocorticoidemia, the HPA axis is controlled by negative feedback. During sepsis, immune cell-derived cytokines such as TNF-α, IL-IL-1-β, IL-6, and IL-10 activate the HPA axis (77). In experimental sepsis, adrenalectomized GRdim/dim mice treated with UX38, a GR blocker, showed poor outcomes (83). Thus, the disruption of the HPA axis plays a vital role in sepsis. Moreover, as in sepsis, higher vascular inflammation results in adrenal and HPA axis dysfunctions (84). Some studies showed that compared to a healthy state, plasma concentrations of total cortisol/corticosterone (CORT) rose in intensive care unit (ICU) septic patients and male mice and rats compared to a control group. However, they also observed reduced serum ACTH levels (85–88). This has been called “ACTH-cortisol disruption.” Likewise, Téblick et al. noticed that pro-opiomelanocortin gene expression was augmented more than usual. However, markers of processing POMC into ACTH and ACTH secretion were downregulated by glucocorticoid receptor–ligand binding. This could explain the low plasma ACTH and high POMC levels (88). Thus, impaired adrenal response during sepsis improves the circulation of FFA, triglycerides, glycerol, gluconeogenic amino acids, and glucose, guiding a starvation response (89, 90).

In conclusion, sepsis triggers a severe stress response that affects both central and peripheral systems, leading to hormonal and metabolic alterations. Disruption of the HPA axis and impaired adrenal response are crucial in sepsis pathophysiology and associated metabolic changes.

## 4 Alterations in lipid metabolism during sepsis

Lipids are the most significant energy reserves in the body, and FAO provides the most ATP total balance, becoming an essential energy source in high metabolic demand scenarios, such as sepsis. Lipids are usually conserved as triglycerides (TGs) in the cytoplasm of adipocytes. In order to be used as energy substrate, TGs suffer lipolysis to form fatty acids and glycerol, a reaction mediated by hormone-sensitive lipase (HSL). (91). Inflammatory stimuli trigger lipolysis via IRE1 kinase (92). Acyl-CoA synthase activates lipids and enters the mitochondria as carnitine derivatives through membrane transporters such as carnitine palmitoyltransferase 1 (CPT1), which is regulated by the nuclear receptors PGC-1α and PPAR-α (93). It has been reported that LPS suppresses FAO by reducing the expression of genes related to essential enzymes in lipid metabolism, including CPT-1, PGC-1α, and PPAR-α (94). Also, in an LPS-induced model, Li and coworkers 2021 (95) showed that CPT-1 and PGC-1α expression were reduced in cardiomyocytes and suggested a strong dependency of Nrf2 as a co-factor of PGC-1α. In addition, CPT1A inhibition is associated with a higher risk of infection and impairment of neutrophil response (96).

Elevated serum glucocorticoid levels promote endocrine and metabolic alterations, leading to a catabolic state, peripheral FAO, and ketogenesis (89, 97). Lpin1, Fabp4, Gpat4, Angptl4, Dgat1, Dgat2, PNPLA2, and LIPE are glucocorticoid-responsive genes that enhance adipose tissue lipolysis (98, 99). In patients with sepsis and sepsis animal models, FFA serum levels are high, and peripheral organs utilize these FFAs to produce energy. FFA also activates and upregulates the expression of PPAR-α, the central transcription factor responsible for inducing the β‐oxidation of FA and the production of ketone bodies (22). However, according to several studies, including human and animal models, PPAR-α levels are downregulated in sepsis, and β‐oxidation is compromised, causing FFA to accumulate in the liver, heart, kidney, and blood (20, 100–102).

Lipotoxicity occurs when lipids accumulate intra- or extracellularly, beyond physiological levels (103). In sepsis, mitochondrial oxidative phosphorylation is affected by the inflammatory state of the host (104). Therefore, this deficit in FFA breakdown during sepsis causes energy shortage, lipotoxicity, and mitochondrial damage due to FFA accumulation. Supplementation with oleic acid increases FAO and protects septic mice from organ dysfunction and mortality (105). On the other hand, FFA can trigger acute respiratory distress syndrome (ARDS) by inhibiting Na/K-ATPase in the lungs, causing tissue damage, edema, and inflammation (106–108).

**Figure 1:**
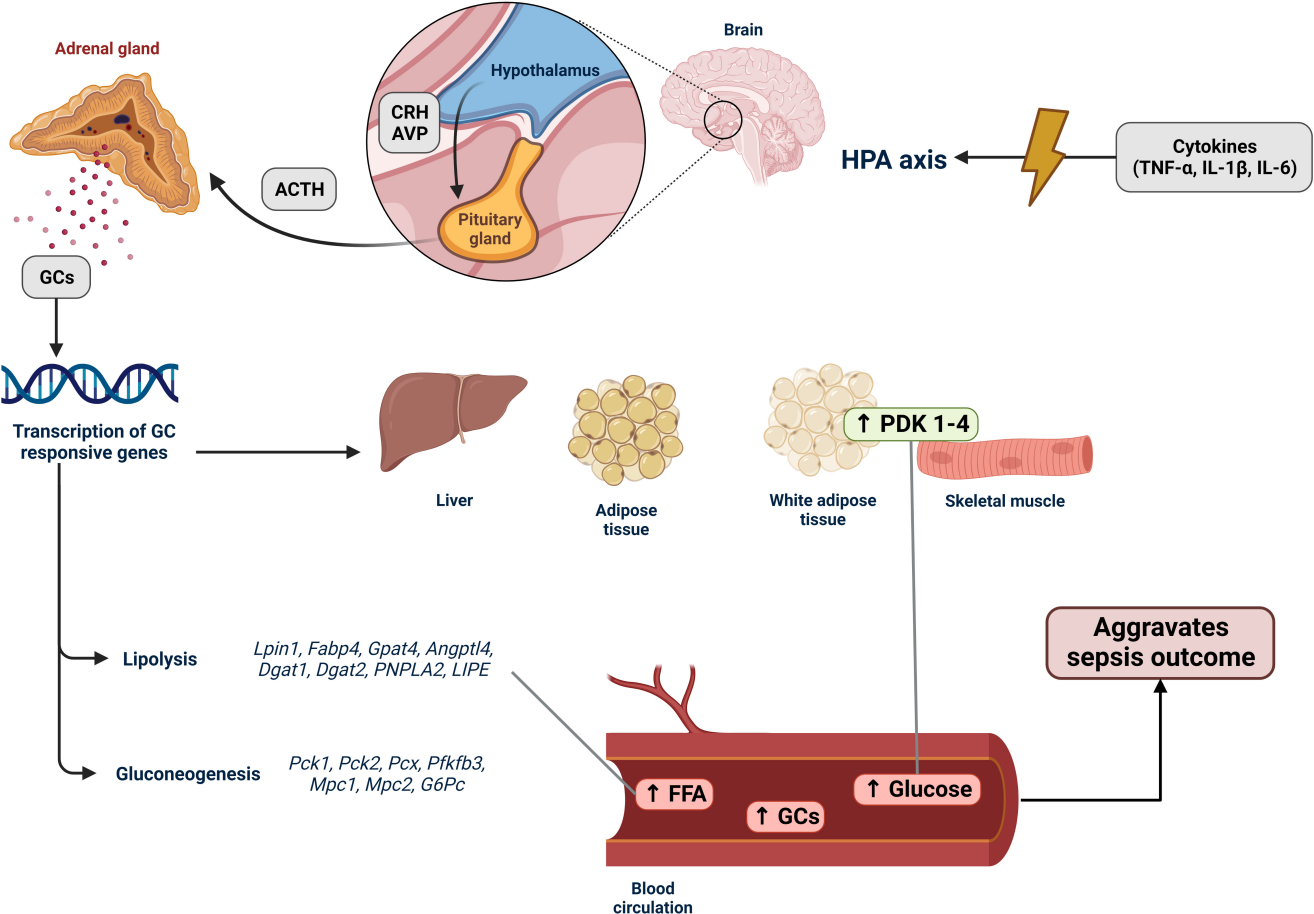
Activation of the HPA axis in sepsis and its effects on lipid metabolism. The hypothalamic–pituitary–adrenal (HPA) axis is an endocrine system that responds to stressful situations. Hormones produced by the HPA axis regulate cell metabolism and immune system functions. In sepsis, an imbalanced immune response leads to an increase in the production and release of various proinflammatory molecules, including tumor necrosis factor-α (TNF-α), interleukin-1β (IL-1β), and interleukin-6 (IL-6). These cytokines stimulate the HPA axis by activating the hypothalamus to secrete corticotropin-releasing hormone (CRH), which in turn stimulates the anterior pituitary gland to produce adrenocorticotropic hormone (ACTH) and arginine vasopressin (AVP). ACTH then promotes the production of glucocorticoids (GCs) in the zona fasciculata of the adrenal cortex. GCs are released into the bloodstream, reaching various tissues, particularly the liver, muscles, and adipose tissues. When coupled with glucocorticoid receptors (GRs), GCs induce transcription of GC-responsive genes. In sepsis, the expression of lipolysis-associated genes (Lpin1, Fabp4, Gpat4, Angptl4, Dgat1, Dgat2, PNPLA2, and LIPE) is upregulated. This upregulation increases the breakdown of fatty acids, resulting in the accumulation of free fatty acids (FFAs) in tissues and the bloodstream, indicating lipotoxicity during sepsis (*Section 4*). Genes associated with gluconeogenesis (Pck1, Pck2, Pcx, Pfkfb3, Mpc1, Mpc2, and G6Pc) were also upregulated, along with the increased expression of PDK1–4. These changes contribute to reduced glucose uptake and the hyperglycemia development. Hence, the HPA axis stress response system regulates the host response to internal and external signals, such as the intense inflammatory state observed in patients with sepsis. Consequently, it has a decisive impact on sepsis because hyperglycemia, accumulation of FFAs, and elevated GC levels are associated with poor patient outcomes.

Additionally, GR promotes the transcription of gluconeogenic genes such as Pck1, Pck2, Pcx, Pfkfb3, Mpc1, Mpc2, and G6Pc (99) by diminishing glucose uptake by suppressing GLUT4 translocation (109) and by reducing glucose utilization in skeletal muscle and white adipose tissue through the upregulation of PDK1 to PDK4 (22, 110). Hence, septic animal models and patients present with hyperglycemia during the acute phase of sepsis (111). Interestingly, patients with sepsis show insulin resistance, which causes an increase in lipid mobilization. In addition, inflammatory signaling elicited by LPS can induce lipolysis (92), further increasing FFA levels. Hyperglycemia, increased FFA levels, and insulin resistance are associated with worse outcomes in sepsis (112) (**Figure 1**).

Saturated fatty acids (SFA) activate the TLR4/MyD88/NF-kB pathway leading to endothelial inflammation *in vitro* upon FFA stimuli, with increased levels of CCL5, IL-6, IL-8, ROS, metalloproteinases (MMP-2 and MMP-9), and adhesion molecules (VCAM-1 and ICAM-1) (113). FFA uptake by cells is mediated by membrane receptors such as cluster of differentiation 36 (CD36), which is a transmembrane glycoprotein and a class B scavenger receptor (SR-B2) widely expressed throughout mammalian tissues (114). FFA can cause kidney lipotoxicity through the PPARγ/CD36 pathway in mice fed a high-fat diet. CD36 was found to be upregulated in the kidneys of obese mice alongside PPARγ, which is a crucial CD36 transcription factor, and inflammatory cytokines such as IL-6 and IL-18 (115). In addition, CD36 has been associated with metabolic diseases that present alterations in lipid metabolism (116) and is upregulated in septic patients (117) and sepsis animal models (118). Furthermore, CD36 deficiency improved sepsis outcomes in mice (119). CD36 plays a rate-limiting role in fatty acid uptake, making it a key regulator of cell lipid utilization.

Cell lipids can be used as substrates to generate oxylipins and other lipid mediators that act on immune cell signaling during inflammation. CPT1 also plays an essential role in transporting oxylipins to the mitochondria during infection, as evidenced by the accumulation of oxylipins in the peritoneal lavage of animals challenged with LPS and CPT1 inhibitors. Similarly, CPT1 controls COX/LOX-derived oxylipin secretion by increasing its uptake into the mitochondria. The authors suggested that CTP1 inhibition dampens the inflammatory response by increasing PGE2 and PGI2 levels (120).

In the face of sepsis, the body’s tissues confront a dire state of reduced oxygen supply, instigating a decline in FAO within the muscles. The liver tries to adapt swiftly by augmenting gluconeogenesis and FAO for energy production (22). However, intensified FAO by the liver can ultimately produce ketonic bodies (121). Moreover, the critical interplay between dwindling oxygen levels and the potential upsurge in FAO applies an unwavering pressure on the respiratory chain, culminating in a cascade of ROS generation (122, 123). Such intricate metabolic changes may have far-reaching implications for the pathogenesis of sepsis and clinical outcomes of affected patients.

Alterations in lipid metabolism are critical contributors to sepsis outcome. Despite being an important energy source, the accumulation of lipids in both the tissue and blood circulation can have harmful effects. In sepsis, the uptake of FFA by cells increases, but its oxidation by mitochondria is impaired, leading to lipotoxicity. FFA originating from augmented lipolysis activates inflammatory signaling pathways, thereby increasing the imbalanced host immune response. The endocrine system also plays a role in lipid metabolism by regulating the expression of lipid utilization genes through the activation of the HPA axis.

## 5 Heart and skeletal muscle metabolism during sepsis

Sepsis-induced myocardial injury is an acute type of cardiomyopathy that is a common complication of sepsis (124, 125). Bacterial exotoxins are primary triggers of the septic heart immune response (126). The pathogenesis of septic cardiomyopathy (SCM) is complex and involves both myocardial and peripheral vascular dysfunctions (124). Heart damage is associated with the left ventricle, particularly reducing systolic and diastolic function and ejection fraction (124, 125). Peripheral vascular injury is associated with endothelial dysfunction, resulting in leukocyte retention and ROS production (124).

The molecular mechanisms responsible for SCM include different pathways, most of which are related to inflammation. PAMP and DAMPs can activate cardiomyocyte TLR, fostering a septic inflammatory response (124, 125). TLRs play crucial roles in the pathophysiology of myocardial sepsis. For instance, a study using post-mortem myocardial cell lines found that TLR2, TLR4, and TLR5 stimulation increased inflammatory cytokines, especially IL-6 (127). In addition, an increased inflammatory response is related to worse ventricular function in a cell model (127). Furthermore, knockout of TLR4 results in decreased mortality and expression of IL-6 and TNF-α, with better global ventricular function in a sepsis animal model (128). Moreover, bacterial toxins are responsible for direct endothelial damage, causing microcirculation impairment (129, 130).

Once PAMPs prompt TLRs, they trigger intracellular signaling resulting in the release of cytokines, including TNF-α and interleukins, with a unique role in IL-1β and IL-18 (124, 125). A significant association between elevated plasma levels of interleukin-8 (IL-8) and cardiac dysfunction was observed in individuals with sepsis-induced myocardial dysfunction (131). TNF-α is also associated with depressed myocardial tissues (132). In addition, SFA can bind and activate TLR to promote tissue inflammation (133, 134).

Nitric oxide (NO) is also associated with cardiac dysfunction in sepsis. Initially, NO helps maintain hemostasis and hemodynamics. However, increased concentrations contribute to the deterioration of heart function, which may contribute to acute heart failure and dysfunction in sepsis (135, 136). Higher levels of NO induce decreased left ventricular contractility in animals treated with exotoxins (137). In addition, a previous study suggested that endothelial dysfunction could be responsible for leukocyte retention in the coronary vasculature (138). This increased leukocyte activity also increases inflammatory cytokine production and contributes to LV dysfunction.

In SCM, myocardial metabolism is impaired. Under physiological conditions, FFA is the preferential energy source of the myocardium. FAO generates approximately 70% of the heart’s adenosine triphosphate (ATP) (139), followed by carbohydrates (140). Cells can also generate ATP from amino acids and ketone bodies (140). However, the ability of heart cells to store high-energy phosphate is poor. Thus, mitochondria play a fundamental role in cellular and energy homeostasis (140).

In sepsis, cardiac metabolism is more dependent on glycolysis and lactate metabolism, and FFA and ketone bodies are reduced (141, 142). Under healthy conditions, FAO is the primary energy source for cardiomyocytes (142). However, a septic heart downregulates FAO and relies on other energy sources (143). The FAO is reduced for the following reasons as follows. FFA uptake by CD36 is impaired because IL-1β downregulates the expression of very-low-density proteins (VLDLs) receptors in cardiomyocytes (142). In addition, TLR-mediated inflammation is responsible for reducing the expression of FFA-binding proteins, acyl-CoA reductase, and FAO-associated transcription factors (142). Among the transcription factors, PPAR and its coactivator are the most essential. In sepsis, reduced expression of PPAR-γ in cardiomyocytes and adipose tissue was associated with lower levels of FAO and ATP production, as well as an increase in triglyceride accumulation, contributing to a general impairment in the heart’s metabolism (105, 139, 142, 144). Other molecular factors associated with decreased intracellular FAO include carnitine palmitoyl transferase-1 and acyl-CoA synthase (145).

Studies suggest that the oxygen supply to the heart is not generally affected during sepsis (142). Hence, it is hypothesized that metabolic changes, especially a reduction in FAO, are related to the genetic background (145). In inflammation, lipoprotein lipase (LpL) activity is markedly reduced, possibly due to the increased expression of Angptl4 (146), an LpL inhibitor. The expression of VLDL and CD36 receptors also decreases (142), compromising lipid uptake by the heart. Even though this causes a negative metabolic response, it is possible that the increased lipid clearance acts as a defense response since bacterial capsules are mainly composed of lipoproteins. The more these structures are removed from the organism, the less toxic the effects they exert (145).

Reduced PPAR expression in sepsis is associated with worse outcomes (147). A previous study by our group discussed the relationship between PPARγ and FAO (148). PPAR plays a significant role in modulating the immune response by regulating and controlling cytokine release (68). The higher the expression of PPARγ, the higher the immune response and its negative impacts. This is true not only for the myocardium but also for other organs. The effects of reduced FAO go further. Decreased oxidation leads to increased levels of non-esterified fatty acids (NEFA), which are also associated with a poorer prognosis in sepsis (105). In addition, increased circulating NEFA levels are associated with hypoalbuminemia and liver failure, which occurs during sepsis (134).

Metabolic changes in SCM also result from mitochondrial dysfunction because the heart is highly dependent on oxygen production (149). After endothelial injury, leukocytes are chemoattracted, increasing ROS production and causing organelle injury, including the mitochondria (150). Harmed mitochondria activate cAMP/protein kinase A signaling and produce more ROS by inhibiting the oxidative phosphorylation complex and lowering oxygen consumption (149). Consequently, this pathway triggers oxidative stress and apoptosis (124). In addition, mitochondrial DN (mtDNA) is essential for regulating immune responses. Studies have shown that individuals with an increased inflammatory response may present with increased mtDNA in their cytoplasm, related to mtDNA release from the organelle (149). Cardiolipin is one of the most critical targets of ROS damage in mitochondria. Cardiolipin causes structural damage to the mitochondria and impairs oxidative phosphorylation (151). Another possible target of ROS damage is poly(ADP-ribose) polymerase (PARP) enzyme, which is involved in DNA repair. This enzyme is upregulated in sepsis and may be associated with mitochondrial structural damage (151).

The Complement System is also linked to ROS damage in SCM, specifically C5a pathway (152, 153). Complement C5a can be activated early in sepsis and contributes to a robust inflammatory response (154). An imbalance in calcium homeostasis, caused by complement C5a and mediated by ROS, can harm cardiomyocytes (155). The activation of MAPks and Akt, which contributes to heart dysfunction in bacterial sepsis, has been linked to complement C5a (153). In addition, the complement system affects coagulation hemostasis, contributing to disseminated intravascular coagulation (DIC) during sepsis (154, 156).

In addition to causing damage to the myocardial muscle, sepsis affects the skeletal muscle, causing fiber wasting and sarcopenia. A meta-analysis by Liu et al. (157) suggested that sarcopenia may predict mortality risk in patients with sepsis. Muscle atrophy is a consequence of increased degradation and decreased repair of skeletal muscle. In addition, the increased secretion of cytokines causes increased activation of proteolytic enzymes, resulting in muscle loss.

The muscle structure and function are based on thinned-regulated homeostasis. Sepsis disrupts skeletal fiber homeostasis. Muscular homeostasis is a complex balance between muscle inflammation, degeneration, fibrosis, repair, and regeneration (158). However, literature suggests that muscle injury promoting regeneration and growth involves a pathophysiological process other than inflammatory wasting (158). Muscle injury and its repair mechanisms are mainly mediated by neutrophils and macrophages (158). After injury, neutrophils migrate to the tissue, and there is an increase in the production of inflammatory cytokines, including TNF-α, IL-1, and INF-γ (159). Specifically, TNF-α activates NF-kB, which causes muscle protein destruction (160). Nitric oxide secretion mediates macrophage degradation (158).

One of the apoptopic mechanisms seen in septic muscle wasting is related to the overwhelming endoplasmic reticulum (ER). In skeletal fibers, the ER, also called the sarcoplasmic reticulum, balances intracellular calcium according to the cell’s needs. ER is also involved in a protein-folding regulation mechanism that activates apoptosis-triggering pathways (161). Overstressed ER accumulates excess folded proteins, which activate three key molecules that participate in the unfolding process: protein kinase R-like endoplasmic reticulum kinase (PERK), inositol requiring protein 1a (IRE1a), and activating transcription factor 6 (ATF6) (161). If this process fails or the cell remains under stress, apoptosis pathways are activated (161). This process activates the ubiquitin–proteasome system and autophagy–lysosomal pathway, triggering cell death via proteases and caspases (161).

During muscle atrophy, contractile proteins are degraded by the ubiquitin-proteasome system (UPS), autophagy–lysosomal pathway (ALP), and proteases such as calpains and caspases (149). Studies suggest that muscle protein degradation follows a two-step pathway in which calpains and caspases first disassemble skeletal fibers, and then, proteases and other proteins from the ubiquitin complex act on the degradation of actin fibers (162). UPS-mediated protein degradation is regulated by a specific group of ubiquitin ligases including atrogin-1 and MuRF1. These enzymes target muscular proteins for ubiquitin-mediated degradation (162). However, this process is controversial because it has not yet been demonstrated *in vivo* (162).

Irisin is an essential protein related to muscle waste. Irisin was first discovered in 2012, and studies have shown that the expression of irisin and its precursors is related to muscular tissues (163). Irisin levels may be depleted during sepsis because of a cytokine storm (164). This is true because irisin modulation chemicals (TLRs, MAPK, NF-kB) are increased in a pro-inflammatory response, such as sepsis or COVID-19. This could explain the role of muscle waste in inflammation-related diseases (164).

Overall, an increased inflammatory response, lower oxygen supply, loss of tissue homeostasis, and cell destruction in the muscle result in decreased FAO and an increase in FFA/NEFA circulating levels.

## 6 Hepatic dysfunction during sepsis

**Figure 2:**
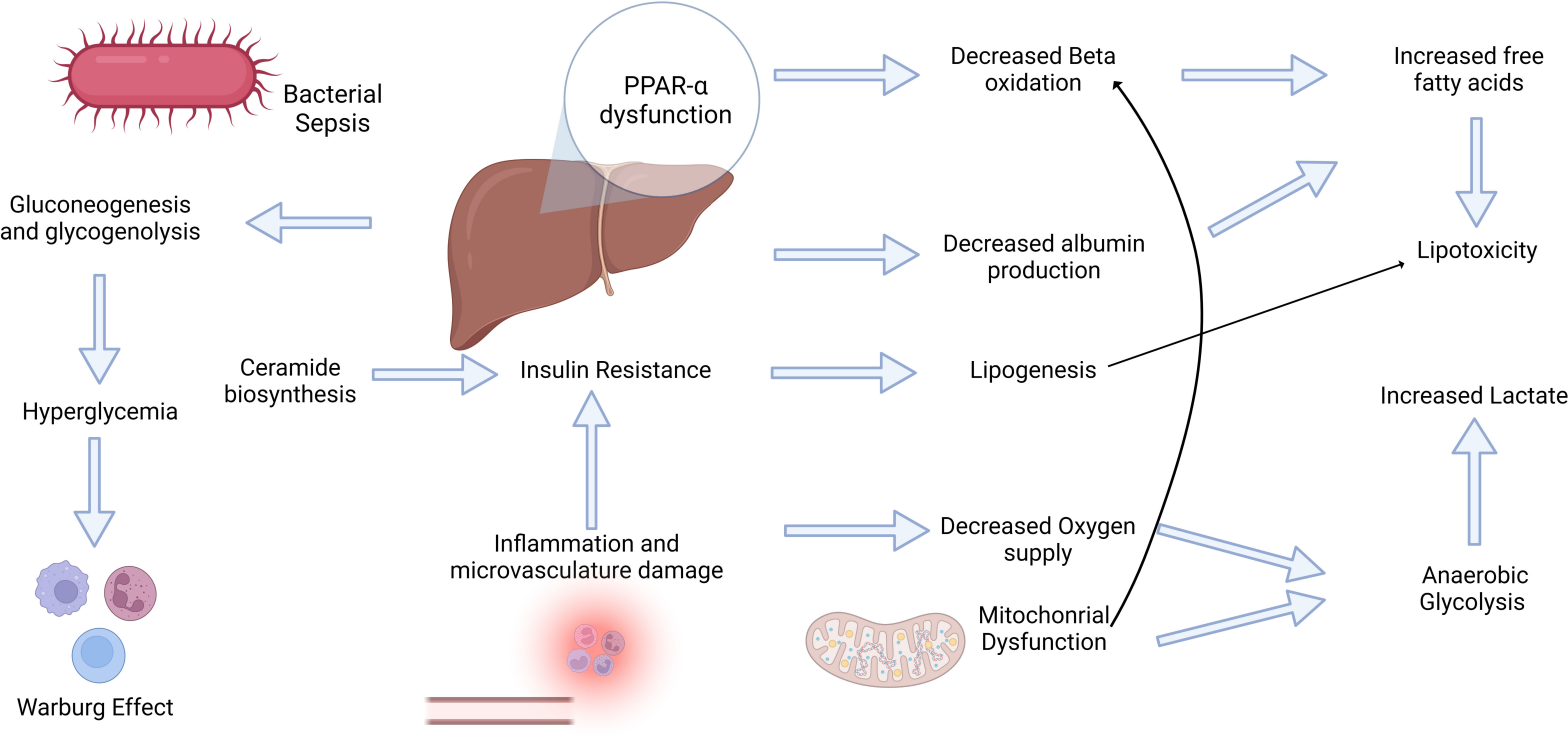
Sepsis induces a hypermetabolic state that triggers a complex interplay between inflammatory and metabolic changes. The bidirectional relationship between inflammation and metabolism plays a pivotal role in the development of sepsis. Inflammation triggers metabolic alterations and vice-versa. Inflammatory mediators, such as free fatty acids, can trigger insulin resistance, which is crucial for subsequent metabolic alterations. Conversely, hyperglycemia may elicit significant effects on immune cell activity, including the Warburg Effect. These multifaceted adaptations and dysfunctional PPAR-α levels contribute to increased oxidative stress, lipotoxicity, and elevated lactate levels, ultimately contributing to progressive organ failure.

The liver is central to human metabolism in health and disease states. The liver is pivotal in orchestrating the acute phase response (APR) in critical illness and systemic inflammation. It releases proteins that participate in essential activities such as coagulation, transport, and immunological functions. (165, 166). In clinical practice, preventing bacterial translocation in the gut is crucial because it can lead to bacteremia and sepsis (167). During translocation, the bacteria enter the hepatic sinusoids through the portal vein. The liver acts as the second line of defense, constituting the gut–liver axis and promoting bacterial clearance (168, 169). Sepsis is a hypermetabolic state wherein the patient’s feeding is often limited and does not supply daily energy requirements; therefore, metabolism mimics a starvation state (170). Thus, the liver promotes metabolic adaptations to sepsis, including alterations in glucose and fatty acid metabolism (**Figure 2**).

Investigating the interplay between metabolic and inflammatory alterations is crucial for understanding the inflammatory conditions (171). The role of inflammation in insulin resistance has been widely accepted in critically ill patients (172) (**Figure 2**). Cytokine signaling may stimulate the inhibitory phosphorylation of insulin receptor substrate 1 (IRS-1) by stress kinases such as JNK1 and IKKb (173). Cytokines may also enhance hepatocyte ceramide production, thereby increasing their production in sepsis (174, 175). Ceramide biosynthesis appears to be involved in insulin resistance (**Figure 2**), and TLR4 stimulation by SFA increase enzymes in this pathway (176–178). Supported mainly by the insulin resistance response, sympathetic stimulation, and limited oral starvation, the liver increases glucose production through gluconeogenesis and glycogenolysis during sepsis. However, glucocorticoid resistance and iron‐driven oxidative inhibition of glucose‐6‐phosphatase may hinder gluconeogenesis in the liver (179, 180). It usually leads to stress hyperglycemia during the acute phase of sepsis and in other critically ill patients, even in non-diabetic patients (181). Extremely high glycemic levels in these patients are associated with poor outcomes and an increased risk of developing type II diabetes (182, 183). However, hyperglycemia may be an adaptive mechanism to sustain immune cell activity because these cells shift their metabolism based primarily on glycolysis with lactate formation, even in an aerobic environment. This phenomenon is known as the Warburg effect, and it was first described in cancer cells; however, it similarly occurs in monocytes, dendritic cells, and T cells during their activation, mediated by increased succinate release into the cytoplasm and Akt-mTOR-HIF-1α (184–186). This metabolic shift may support the elimination of ROS and development of trained immunity (187, 188). However, in tissue ischemia, this phenomenon leads to an increased release of lactate. Lactate levels are correlated with adverse outcomes in patients with sepsis. They can activate TLR4, promoting the activation of the NF-kB pathway and release of inflammatory mediators (189). In addition, the increased accumulation of succinate may trigger inflammation via ROS generation mediated by succinate dehydrogenase (190, 191).

In addition, adipose tissue provides energy storage in mammals, releasing fatty acids that can be oxidized to produce ATP and maintain cell activities. It has been reported that lipolysis is upregulated during sepsis, increasing plasma levels of fatty acids and triglycerides (192). LPS challenge induces the enzymatic activation of hormone-sensitive lipase (HSL) by phosphorylation at Ser650 and an increase in perilipin-1 phosphorylation by protein kinase A (193). Insulin resistance in these patients also supports lipolysis and an increase in catabolic hormones, such as adrenaline, GH, and glucagon (194–196). Depressed lipoprotein lipase activity has also been postulated to cause hypertriglyceridemia, occurring not necessarily in sepsis but in endotoxemic and bacteremic states (197). It is worth highlighting that the shift in lipid metabolism can support even the conceptualization of new prognostic tools. Modified hepatic levels of selected phospholipids have been linked to impaired energy metabolism and the progression of sepsis (198). A recent metabolomic study has suggested differences in plasma lipid concentrations between survivors and non-survivors, medium/short-chain hydroxy acyl-CoA dehydrogenase (elevated in survivors), and crotonase (elevated in non-survivors). Hence, there is a future perspective for monitoring cellular energy beyond the currently established methods (199).

Unfavorable outcomes in sepsis are related to impaired metabolic adaptation in the liver (200). PPAR-α (encoded by the NR1C1 gene) is a crucial transcription factor that regulates gene transcription (e.g., CPT1A and FABP1) of proteins involved in β-oxidation (201, 202). During sepsis and other inflammatory conditions, PPAR-α expression is downregulated in the liver, thereby diminishing lipid oxidation in this organ (20). Malnourishment, which is common in patients with sepsis, is also associated with decreased PPAR-α levels (203). In addition, the entry of fatty acids into mitochondria is partially hindered by the increased concentration of malonyl-CoA generated from glucose, especially in the heart, muscles, and liver (204). In this context, if unable to capture and oxidize lipids, the liver does not shift to a starvation response properly, resulting in adverse outcomes for the patients. A recent transcriptomic study found that PPAR-α-deficient mice presented more severe glycemic disturbances and increased steatosis than control mice (205). Ketogenesis may also be impaired, which can be fatal after LPS-induced endotoxemia (206). Ketone bodies can protect against ROS and provide energy to extrahepatic tissues (207). Ultimately, downregulation of PPAR-α-dependent genes impairs liver metabolic adaptation in sepsis (205). Conversely, PPAR-α agonists may benefit septic patients (20, 208, 209).

Potential therapeutic approaches for dysfunctional lipid metabolism during sepsis, agonists of peroxisome proliferator-activated receptors α and γ, and omega-3 fatty acid supplementation have shown promise (210, 211). Saroglitazar (SAR), a dual PPAR-α/γ agonist, has anti-inflammatory and antioxidant effects that mitigate LPS-induced liver and kidney injury during sepsis (212). PPAR-α activation counters lipogenesis, endotoxemia, and dysbiosis, ameliorating the intestinal barrier structure and reducing TLR4 expression in the livers of high-fructose-fed C57BL/6 mice (213). In addition, PPAR activation by fenofibrate showed protective effects against cardiac damage in septic mice by reducing troponin-T, ROS, and IL-6 TNF-α (214). Rosiglitazone, another PPARγ agonist, mitigated apoptosis, and pro-inflammatory responses in LPS-stimulated myocardial cells (215). PPAR agonists improve sepsis-related conditions beyond the muscles and liver (216). A meta-analysis conducted in 2020 with 1,514 patients suggested that omega-3 fatty acid, a PPAR agonist, might be associated with decreased mortality in individuals with sepsis (217). Likewise, omega-3 fatty acids in severe COVID-19 patients with sepsis showed potential benefits, including reduced procalcitonin and IL-6 levels (218). Finally, intravenous omega-3 fatty acid lipid emulsion reduces systemic inflammation, endotoxemia, and sepsis in patients with acute liver failure (219).

In addition, liver metabolic adaptation is hindered by microcirculatory failure. Liver sinusoid endothelial capillaries (LSECs) regulate the immune response and blood flow (220). However, LSEC may be damaged during sepsis, contributing to liver failure. The architecture of LSECs is susceptible to LPS injection, leading to reduced flow velocity, increased heterogeneity, and blood perfusion deficits (221). In mice, toxin levels have been shown to alter the LSEC fenestrae diameter, and the direct effects of LPS and TNF-α can induce hepatocyte damage (222). Different substances have been linked to impaired liver microcirculation, including endothelin-1, carbon monoxide, and nitric oxide (223). In addition, hyperactivation of Kupffer cells (liver-resident macrophages) during sepsis may play a role in hepatic dysfunction and LSEC damage. Kupffer cells can intensely release ROS, TNFα, and IL-1β, leading to oxidative stress and damage to cellular structures, while recruiting other immune cells to the liver (224). Additionally, Kupffer cells can stimulate coagulation, forming microthrombi that further exacerbate LSEC damage (225). A mouse model has revealed that Kupffer cells can contribute to the onset of intrasinusoidal thrombosis, leading to acute liver failure (226). Therefore, oxygen supply mismatch in hepatocytes can impair aerobic energy production, leading to cell damage or death.

As described, sepsis patients present with increased lipid levels in the plasma and tissues due to stimulated lipolysis, impaired beta-oxidation, and hampered transport into the mitochondria. In addition, LPS and cytokines such as IL-6 and TNF-α may induce *de novo* synthesis of lipids, regulated by sterol regulatory element-binding proteins (SREBP) (227). High levels of insulinemia seem to increase SREBP expression in the hepatocytes. A recent transcriptomic investigation found upregulation of ERLIN1 (gene regulating SREBP) during sepsis (228, 229). In addition, LPS administration in murine livers upregulates SREBP-1 (230). Because of the production of toxic metabolites, mainly through lipid peroxidation, increased lipid levels can harm patients, causing cell damage and apoptosis, a phenomenon known as lipotoxicity (37). The double bonds of polyunsaturated fatty acids are major substrates for oxidization by free radicals. Sepsis induces the formation of many peroxidation products such as malondialdehyde (MDA) and 4‐hydroxynonenal (4‐HNE), which lead to toxicity by interacting with amino acids and nucleosides (231, 232). Additionally, diacylglycerol, ceramide, palmitate, and other SFA can be upregulated in sepsis, resulting in lipotoxic effects (233–236). SFA can promote inflammation mediated by TLR-4, while TLR-4 knockout mice may not suffer from SFA signaling (237). Possible therapeutic targets to prevent lipid peroxidation and lipotoxic effects in patients have been investigated. Administration of C75, a fatty acid synthase inhibitor, reduces inflammation and organ injury in sepsis (238). In addition, mitochondrial uncoupling proteins, such as UCP3, have been postulated to function in the defense against lipid-induced oxidative muscle damage (239, 240). Moreover, propofol, a common drug used to manage critically ill ICU patients, may protect against hepatic lipid peroxidation, oxidative stress, and inflammation (241–243).

In addition, albumin synthesis in the liver is decreased by sepsis-related liver failure and general inflammation, making albumin a negative acute-phase protein (244, 245). Serum albumin levels are decreased in most patients with sepsis, although it remains unclear whether this is due to suppressed albumin production or increased albumin clearance (246). Albumin is a crucial fatty acid transporter in systemic circulation. This decrease also favors increased plasma levels of fatty acids (247, 248). In addition, NEFA can activate toll-like receptors and inhibit Na+/K+-ATPase, causing lung injury and edema (249, 250). Our group showed a relationship between the oleic acid/albumin molar ratio and clinical outcomes in critically ill patients, with a higher ratio indicating higher mortality in these patients (251). Omega-9 treatment increases CPT1A mRNA in the livers of septic mice, reduces plasma NEFA levels, and improves survival and clinical status (105). CPT1A plays a crucial role in importing fatty acids into the mitochondrion, delivering them to their final destination oxidation, thus pointing out a possible therapeutic target related to ß-oxidation.

## 7 Study limitations

Sepsis can originate from different microbes, with infections originating in different organs. Here, we focused on bacterial infections and multiple foci of infection. Sepsis severity can influence lipid metabolism. Sex, age, metabolic status, pathological background, and alimentary status may influence lipid metabolism and sepsis outcomes. However, altered lipid metabolism in patients with sepsis has been described, regardless of different conditions. FFA will affect inflammatory and metabolic conditions, which may lead to poor outcomes.

## 8 Concluding remarks

Sepsis is a complex syndrome with a high morbidity and mortality rate. Sepsis pathogenesis disrupts metabolic and inflammatory systems. Derangement of the inflammatory response and metabolic alterations have been identified as targets for the diagnosis and treatment of patients with sepsis. We suggest a new protagonist in sepsis physiopathology, fatty acid oxidation, because FAO in the tissues is reduced and fatty acid blood levels are high. In addition, increased FFA levels are associated with poor prognosis in patients with sepsis. Different mechanisms account for alterations in lipid metabolism during sepsis, such as increased gluconeogenesis and lipolysis, lower oxygen supply to the tissues, increased inflammation, and worsening lipid oxidation. Hence, FAO and FFA can be potentially valuable markers for sepsis diagnosis and prognosis because alterations in their circulating levels and metabolism have a life-threatening impact on critically ill patients.

## Author contributions

CG-d-A conceived and supervised the confection of the whole manuscript. RM-S, GA, MA, IS, MB, and CG-d-A wrote and revised the manuscript. CA and AS critically revised and approved the final version of the manuscript. All authors contributed to the article and approved the submitted version.
